# Supplementary material for: Molecular Dynamics Assessment of Mechanical Properties of the Thin Filaments in Cardiac Muscle
Source: Int J Mol Sci. 2023 Mar 1;24(5):4792. doi: 10.3390/ijms24054792 (PMC10003134; doi:10.3390/ijms24054792)
Supplement: Supplementary file 1 [file ijms-24-04792-s001.zip › ijms-2208540-supplementary.pdf]

Supplementary Materials

Molecular dynamics assessment of mechanical properties of the thin filaments in cardiac muscle

Natalia A. Koubassova, Andrey K. Tsaturyan

Model 2

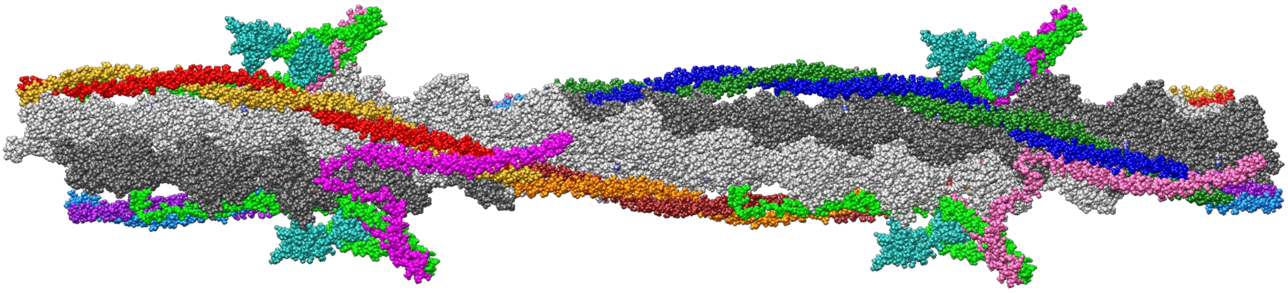

Figure S1. A refined atomic model (Model 2) of a segment of a thin filament of cardiac muscle. The pointed end of F-actin is on the left, *z*-axis in the model coincides with F-actin axis and it is directed from left to right. Actin is shown in light gray and dark gray (long pseudo-helices 1 and 2 respectively), strand 1 of Tpm is red and light brown or brown and orange; strand 2 is light blue and purple or blue and dark green; Tn-C is sea green, Tn-I is light green; two structurally different Tn-T are hot pink (Tn-T1) and magenta (Tn-T2).

Azimutal fluctuations of the tropomyosin chains relative to the long actin helices

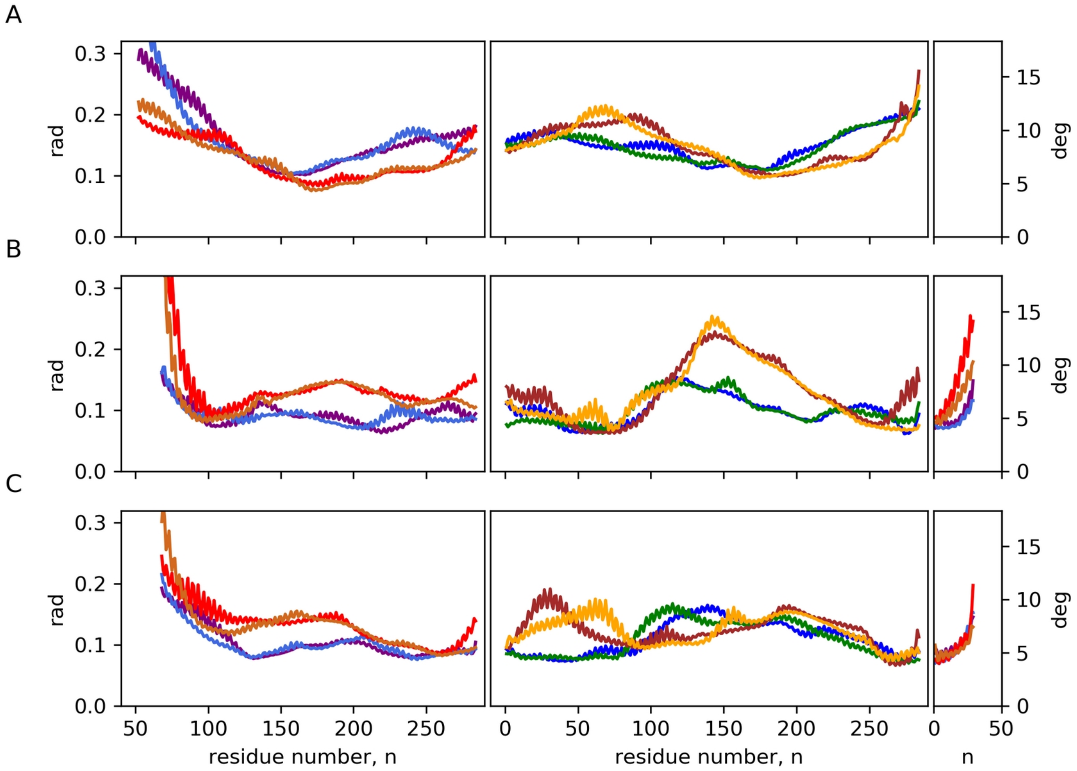

Figure S2. Time-averaged angular fluctuations, SD, of Tpm strands 1 (red/light brown and brown/orange) and 2 (light blue/purple and green/blue) with respect to long actin helices 1 and 2, respectively, shown vs Tpm residue number for each chain in the Model. Color code of Tpm chains is that the same as in Fig 1 and Fig S1. Same data as in Fig 3 in the main text. Upper panel (**A**): Model1, FA+Tpm+Tn, middle panel (**B**): Model2: FA+Tpm+Tn, lower panel(**C**): Model2: FA+Tpm.

Azimuthal fluctuations of the tropomyosin chains relative to the F-actin axes

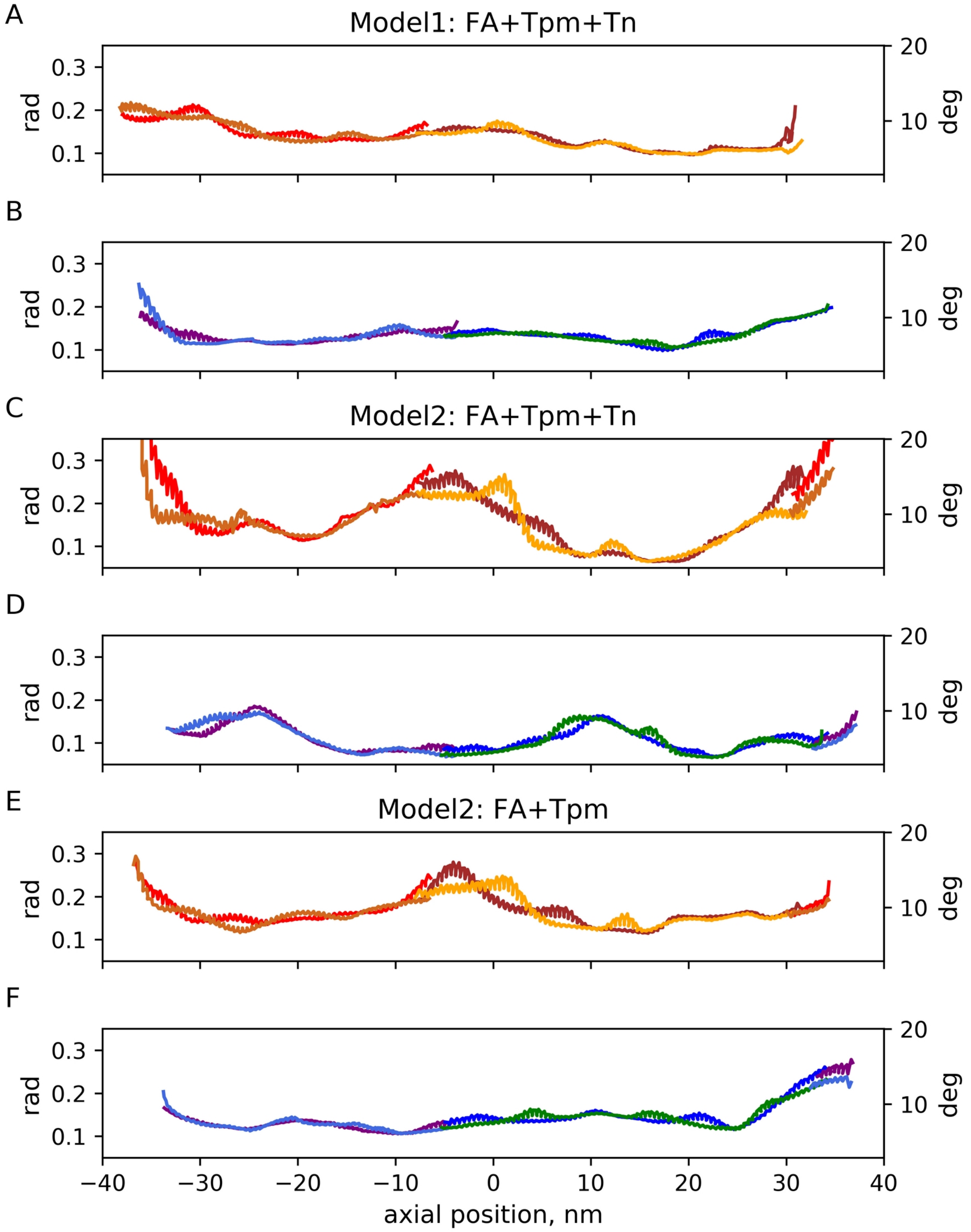

Figure S3. Time-averaged angular fluctuations, SD, of Tpm strands 1 (red/light brown and brown/orange; (**A**, **C**, and **E**) panels) and 2 (light blue/purple and green/blue; (**B**, **D**, and **F**) panels) with respect to F-actin axes.

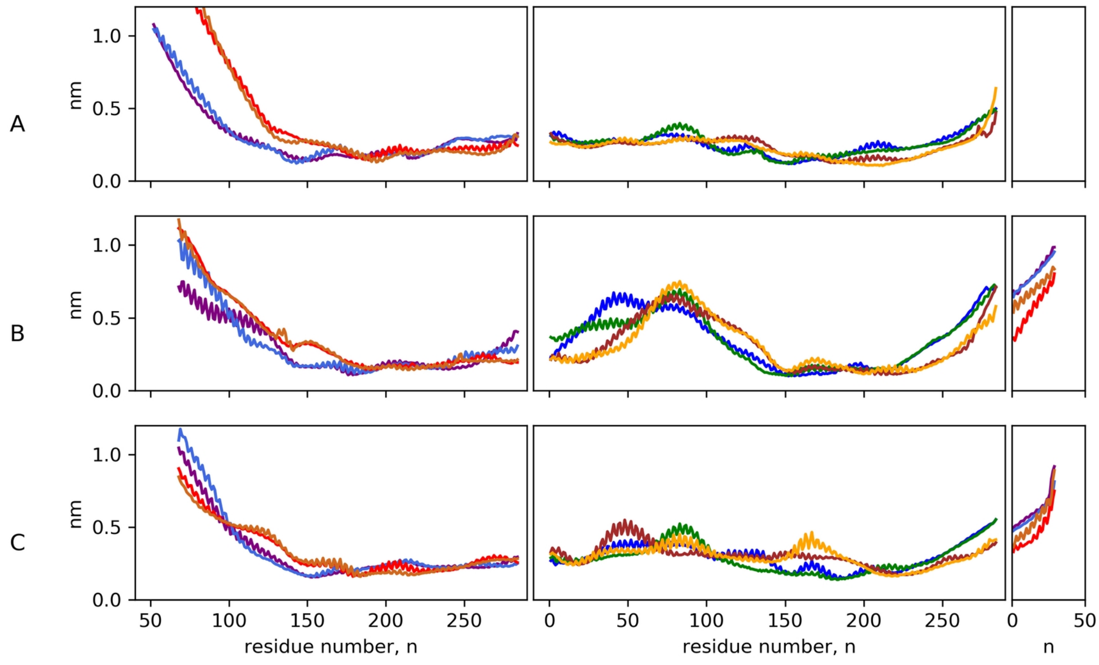

Figure S4. Time-averaged angular fluctuations, SD, of Tpm strands 1 (red/light brown and brown/orange) and 2 (light blue/purple and green/blue) with respect to F-actin axes re-drawn vs Tpm residue number. The same data as in Fig. S3, same sequence of panels. Upper panel (**A**): Model1: FA+Tpm+Tn, middle panel (**B**): Model2: FA+Tpm+Tn, lower panel(**C**): Model2: FA+Tpm.

<u>*Radial fluctuations of the tropomyosin chains*</u>

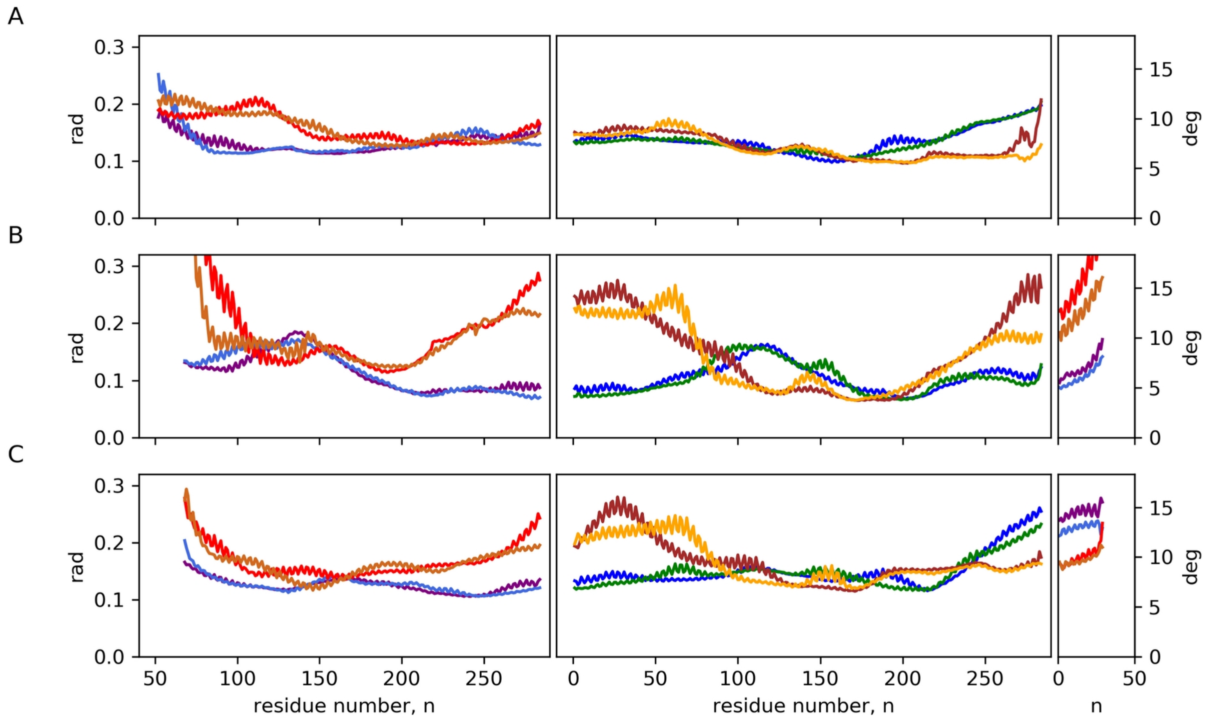

Figure S5. Time-averaged radial fluctuations of Tpm strands 1 and 2 shown in Fig. 4 are re-drawn vs Tpm residue number for each Tpm chain in the models. Color code and data is the same as in Fig. 3 in the main text. Upper panel (**A**): Model1: FA+Tpm+Tn, middle panel (**B**): Model2: FA+Tpm+Tn, lower panel(**C**): Model2: FA+Tpm.

Radial positions of the tropomyosin chains

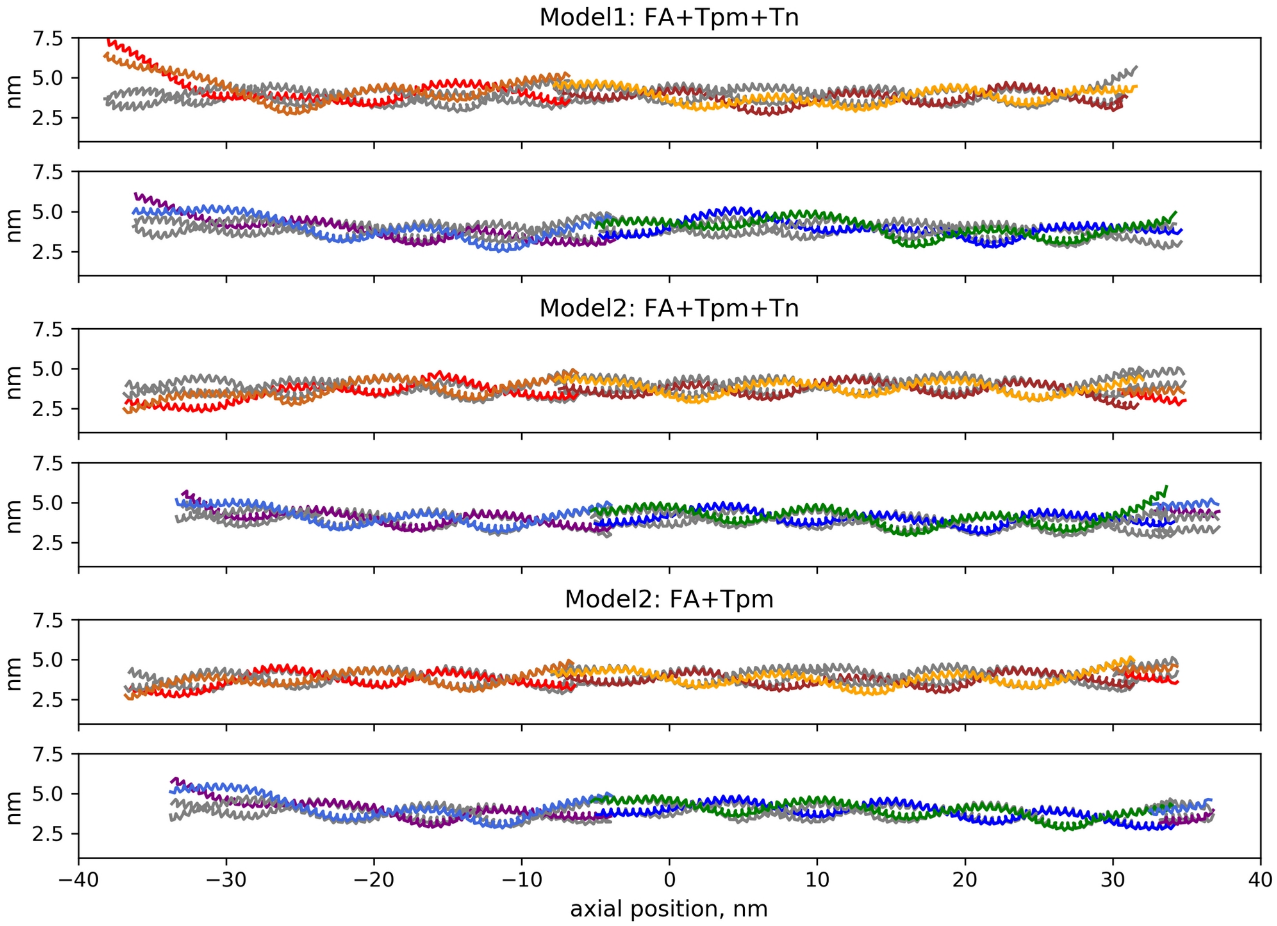

Figure S6. The radial positions of C_α atoms of the Tpm residues in the Models in the first time frame of the MD simulations (t = 0, gray) and their time-average values (red and light brown – the first Tpm molecule; brown and orange – the second one) during three MD trajectories. Color code is the same as in Fig. 1 and Fig. S1.

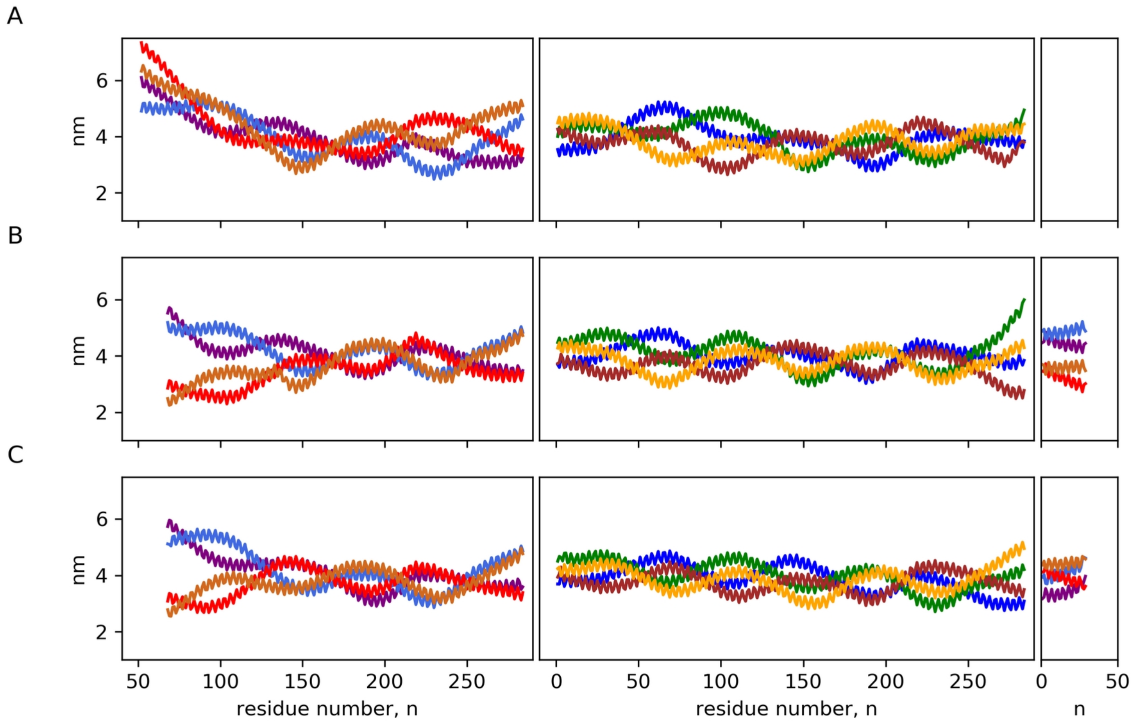

Figure S7. The time-averaged radial positions of C_α atoms of the Tpm residues in the Models (red and light brown – the first Tpm molecule; brown and orange – the second one in the 1st strand; purple and light blue– the first Tpm molecule; blue and green – the second one in the 2nd strand) during three MD trajectories. Same data as in Fig. S6 shown vs residue number, same color code. Upper panel (**A**): Model1: FA+Tpm+Tn, middle panel (**B**): Model2: FA+Tpm+Tn, lower panel(**C**): Model2: FA+Tpm.

H-bonds

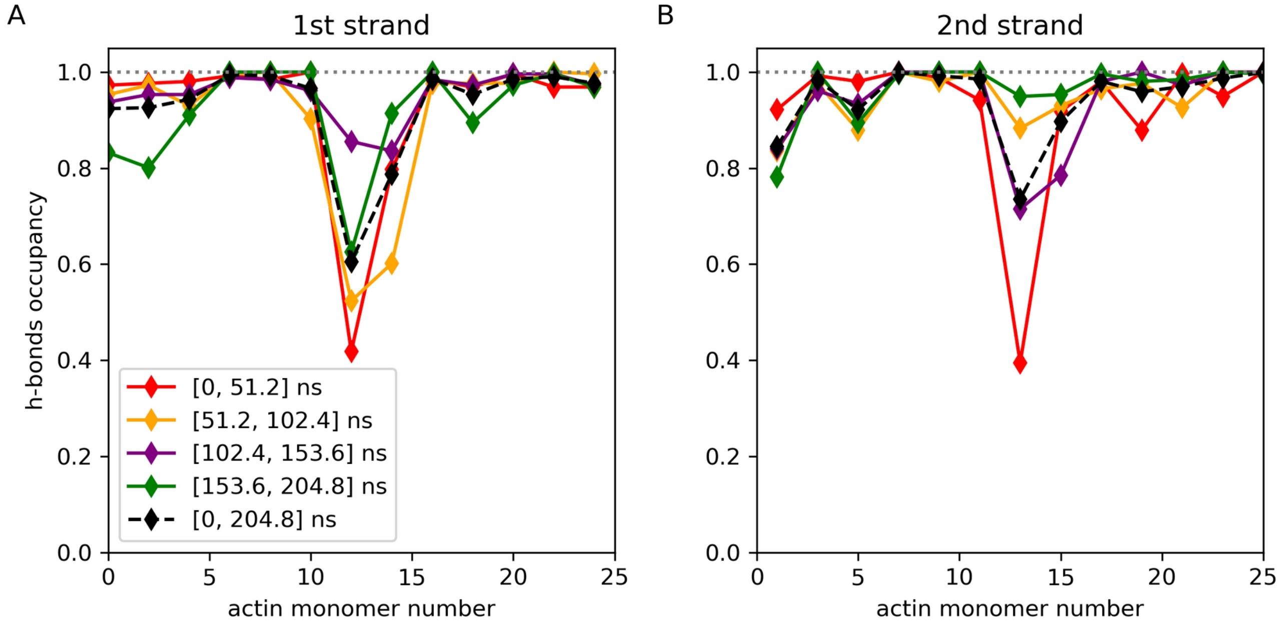

Figure S8. The relative lifetime of the existence of h-bonds between actin residues Lys326 or Lys328 of the long actin helices 1 (**A**) and 2 (**B**) and conserved acid Tpm residues from the strands 1 and 2, respectively, vs number of the actin monomer in the Model 2: FA + Tpm (blue in Fig. 5 of the main text). Data is shown time-averaged by the quarters of the long 204.8 ns MD run together with the time-averaged over whole trajectory values (black dashed line).
